# Biology of Immunoglobulins

**Published:** 2014-12-19

**Authors:** Giorgio Berlot, Perla Rossini, Federica Turchet

**Affiliations:** Dept. Of Anesthesia, Intensive Care and Pain Therapy University of Trieste

**Keywords:** sepsis, septic shock, immunoglobulins, infections

## Abstract

Intravenous Immunoglobulins (IvIg) are often administered to critically ill patients more as an act of faith than on the basis of relevant clinical studies. This particularly applies to the treatment of sepsis in adult patients, in whom the current guidelines even recommend against their use, despite that many studies demonstrated either their beneficial effects in different subsets of patients and that some preparations of IvIg are more effective than other.

The biology of Ig are reviewed, aiming to a more in-depth understanding of their properties in order to clarify their possible indications in different clinical settings.

## 1. Introduction

Intravenous immunoglobulins (IvIg) are currently used in multiple pathologic conditions but they are often prescribed off-label due the absence of specific guidance formulated according to evidence-based medicine (EBM) criteria [1]. This particularly applies to patients admitted to the intensive care unit (ICU), where IvIg may be used either to boost the patients’ immunological capabilities or, conversely, to blunt an immune response directed toward the patients’ own tissues (for example, in myasthenia gravis or Guillan-Barrè syndrome). The uncertainty upon their effectiveness in critically ill patients is further underscored by the recommendations against the use of IvIg reported on the recently issued guidelines of the Surviving Sepsis Campaign (SSC) [2].

The aim of this review is to provide a detailed overview about the biological role of immunoglobulins in relationship with their therapeutic use among critically ill septic patients admitted to the ICU.

## 2. Structures and function of immunoglobulins

**Figure 1:**
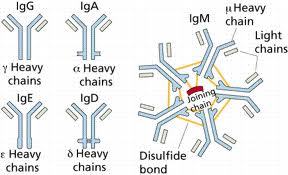
schematic representation of the different classes of Ig.

The ultimate mission of the immune system is to recognize and destroy extraneous molecules invading the host. To be inactivated, a foreign substance must react with fixed or circulating receptors, which trigger the final response. This task is accomplished by two distinct but strictly co-operating systems [3,4]. The innate immune system includes the cells of reticuloendothelial system (RES), the mediators produced and released by these cells during the interaction between the hots and the invading organism and the complement cascade. The number of receptors present on the surface of innate immune system cells is genetically determined and, albeit numerically relevant, cannot match the huge variability of microbial antigenic epitopes. Thus, a more flexible system is required in order to face the myriad of agents and/or substances that come into contact with the host. This second mechanism, known as adaptive immunity due to its capability to cope with continuously changing antigens, involves Ig, which are encoded by genes that are able to undergo somatic recombination and hypermutation. Ig are secreted by plasma cells, which are derived from B lymphocytes that are activated by trapping antigens on a cell-surface receptor and stimulation with CD4^+^ T lymphocytes. Antibodies belong to five different classes of Ig (G, A, M, E, and D). (Figure 1)

**Figure 2.**
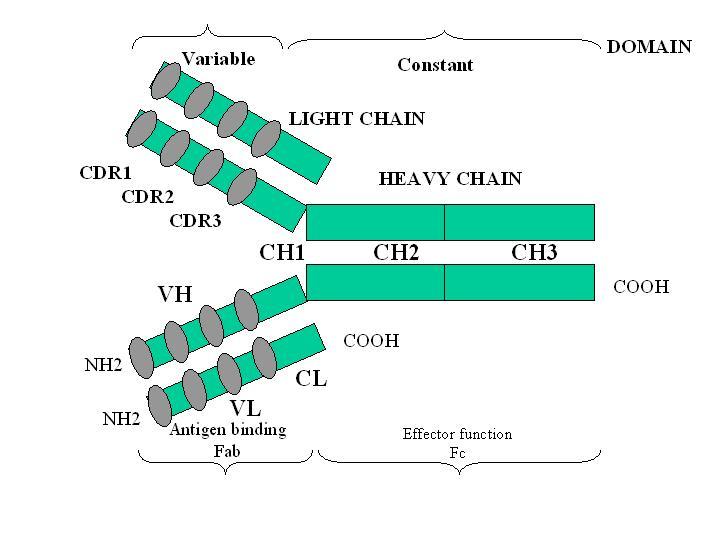
Schematic two-dimensional structure of an IgG molecule. VH and VL indicate the variable regions of the heavy and light chains, respectively. The different epitopes are recognized by the variable regions located on both the light and heavy chains (Fab region). The CDR segments are hypervariable domains located in the Fab regions, which are separated from each other by relatively constant polypeptide chains. The Fc region binds to complement and to the receptors located on the surface of the RES cells and triggers their activation. Legend: CDR: complementary determining region (hypervariable regions); COOH: carboxylic terminal region; C: constant; Fab: fragment antigen binding; Fc: fragment crystallizable region; H: heavy; IgG: immunoglobulin G; L: light; NH_2_: aminic terminal region;; V: variable.

The IgG class is considered the prototypical structure, and consists of a Y-shaped molecule comprised of two identical heavy (H) and light (L) peptide chains. Both H and L chains are divided into a variable (V) domain that reacts with the antigen, and a constant (C) region that activates the various components of the innate immune system, triggering a response (for example, phagocytosis, antibody-mediated and cell-mediated cytotoxicity, and complement-mediated lysis) (Figure 2). The V regions contains three hypervariable regions which are the ultimate responsible of the specific of each molecule of Ig. The H and L regions are linked together by electrostatic forces in association with disulphuric bridges.

The region connecting the two functional parts can undergo conformational changes in order to re-shape the molecule according to the antigen variability.

Therefore, Ig can be considered biochemical transducers able to exert many different actions (Table 1)

Although these actions can justify the administration of IvIg in circumstances characterized by the depression of the immunitary capabilities, yet they are widely used in other disease determined by the production of autoantibodies directed against the patients’s own tissues, such as the myasthenia gravis, the Gullan-Barrè syndrome etc. These opposing indications are a result of the pleiotropic effects exerted by the Ig on the immune system, which include either the augmentation of the immune response through the above described mechanisms but also the down regulation of the inflammatory response via the reduced production of tumor necrosis factor-(TNF-α) and other inflammatory mediators and the increased release of soluble receptors for a number of cytokines [5–7]. This dual IvIg-mediated effects on the inflammatory response suggest that they may be suitable for the treatment of sepsis, which can be characterized initially by an excessive production of inflammatory mediators which can be followed in a later stage by the overall reduction of the immune response [8–9], ultimately leading to an immunoparalysis. Besides the well recognized circumstances associated with the down-regulations of the immunitary response, including the AIDS, the administration of immunosuppressant agents etc, this hypo responsive state characterizes the ICU clinical course of frail patients with multiple underlying pathological conditions, who often survive the initial insult (e.g. pneumonia, emergency surgical interventions etc) but fail to recovery and succumb many weeks after the admission.

An increasing number of mediators are involved in this two-step process which are linked by a complicated array of positive and negative feedback loops [8–10].

Despite the recommendations of the SCC, a number of investigations demonstrated that (a) the administration of IvIg are associated with the reduction of the mortality of patients with sepsis and sepsis-related conditions [11–14],; and (b) IvIg preparations containing IgM and IgA are more effective than those containing IgG alone [15–18].

## 3. The Case of IgM

As stated above, patients given IvIg containing elevated concentrations of IgM have an improved survival as compared with the control and those treated with IgG independently from the age, the underlying conditions and the infecting germ. Although the effect can be ascribed to multiple factors, including the timing of administration and the correctness of the other therapeutic actions, it is hypothesizable that IgM supplementation *per se* can play some role in the improved survival, as it has been demonstrated that plasma values of IgM are reduced in severe sepsis and septic shock and this reduction appears to be more marked and persisting in nonsurvivors [19,20]. Then, on the basis of these observations, it is worthwhile to describe with more details the biological properties of IgM.

IgM is the first antibody produce during an infection, appears first during ontogenesis and has been found throughout all classes of vertebrates [21]. It can exist in a dimeric form on the surface of the membrane of the B cells and circulates as a pentamer (occasionally as a hexamer) in the blood. Its unique structure allows IgM molecules to form strong interactions with different ligands, and has a an extremely higher affinity for the complement than IgG. Experimentally, IgM allows the clearance of apoptotic cells in the immunitary cells in the peritoneal macrophages, and the process requires the activation of Complement. Although it is not yet clear which subset of B cells account for its production, it appears that both B1 and B3 B cells are extensively involved [21]. The role played by IgM during bacterial, viral and fungal infections has been enlightened by studies performed in IgM-deficient mice. On the basis of these observations, it is likely that the circulating pentameric IgM molecules bind ligands more avidly than those present on the cell surface; it is not known if, in the presence of reduced blood IgM concentrations, their role could be replaced by these latter [21]. Surprisingly enough, less it is know about the kinetics as well as the precise therapeutic role IgM in humans. Besides the above quoted investigations which demonstrated that IgM concentrations are decreased in septic shock patients and particularly in those with a poor prognosis [19,20], it appears that reduced levels of this molecule in association with diminished number of natural killer cells (< 58 mg/dl and 140 cell/ml, respectively) are associated with an increased risk of death also in non-septic critically ill patients. Should this findings be confirmed in other studies, the supplementation of IgM could be indicated in life-threatening conditions other than sepsis [22].

## 4. Conclusions

Currently, the administration of IvIg represent the easiest, fastest and less harmful way to interfere with the immunitary system of critically ill patients. Due to their multiple effects, IvIg can be used to boost the response to an infection, to down-regulate an excessive inflammatory response and to abate an autoimmunitary process. The basic knowledge of the biology of Ig is warranted to better individuate the pathophysiologic conditions in whom their use is most valuable. Moving from the lab to the clinical arena, future clinical studies should be addressed toward some relatively unknown aspects of preparations containing IgM, including their T/2, the dose-response curve and their interaction with the antibiotics.

## Figures and Tables

**Table 1.** Possible mechanisms of action of immunoglobulins

**Toxin inactivation** Neutralization of endotoxin and exotoxins Increase clearance of endotoxin Reduce bacterial cell adherence, invasion, and migration
**Stimulation of the leukocyte and serum bactericidal action** Enhancement of endotoxin-induced neutrophil oxidative burst (7S-IvIgG); intact Reduction of endotoxin-induced neutrophil oxidative burst (5S-IvIgG; F(ab’)2 fragments and IgM) Enhancement of serum opsonic activity
**Modulation of cytokine effect** Modulation of the release of cytokine and their antagonists ↓ Pro-inflammatory mediators ↑ Anti-inflammatory mediators Infusion of cytokines and antagonists contained in the Ig preparations Cytokine neutralization by anti-cytokine antibodies
**Modulation of the complement cascade**
